# Microsocial analysis of dyadic interactions with toddlers and mothers with borderline personality disorder

**DOI:** 10.1007/s00737-023-01346-9

**Published:** 2023-07-12

**Authors:** Isabella Schneider, Anna Fuchs, Sabine C. Herpertz, Frances M. Lobo

**Affiliations:** 1 Department of General Psychiatry, Center for Psychosocial Medicine, Heidelberg University Hospital, Voßstr. 4, 69115 Heidelberg, Germany; 2 Department of Child and Adolescent Psychiatry, Center for Psychosocial Medicine, Heidelberg University Hospital, Blumenstr. 8, 69115 Heidelberg, Germany; 3 Department of Psychology, The University of North Carolina at Greensboro, 294 Eberhart Building, Greensboro, NC 27402 USA

**Keywords:** Maternal, Regulation, Synchrony, Contingencies, Non-verbal behavior

## Abstract

Borderline personality disorder (BPD) is known for disruptions in mother-child interaction, but possible underlying patterns of micro-behavior are barely understood. This is the first study examining behavioral dyadic synchrony—the coordinated and reciprocal adaptation of behavior—and regulation on a micro-level and relating it to macro-behavior in mothers with BPD and their toddlers. Twenty-five mothers with BPD and 29 healthy mothers participated with their 18- to 36-month-old toddlers in a frustration-inducing paradigm. Mother and toddler behavior was continuously micro-coded for gaze, affect, and vocalization. Synchrony, operationalized as the simultaneous engagement in social gaze and positive affect, and (co-)regulative behaviors and their contingencies were analyzed and associated with borderline symptom severity, the overall quality of interaction, and child internalizing and externalizing behavioral problems. Dyads with mothers with BPD showed significantly less synchrony compared to dyads with healthy mothers. Low synchrony was associated with high BPD symptom severity and low overall interaction quality. Dyads with BPD used the same amount of regulative behaviors as dyads with healthy mothers. Though both groups equally responded to children’s negative emotionality, mothers with BPD were less effective in drawing the dyad back into synchrony. For dyads with BPD, regulative behaviors were negatively associated with child externalizing behaviors. BPD symptomology may reduce the effectiveness of mothers’ attempts to attune to their child’s needs. An emphasis on synchrony and regulative behaviors may be an important therapeutic target for parenting programs in mothers with BPD.

**Supplementary Information:**

The online version contains supplementary material available at 10.1007/s00737-023-01346-9.

## Introduction

Positive mother-child interactions play a central role in the quality of the mother-child relationship, child development, and the mother’s experience of parenting (Feldman et al. 2011b). The dynamic, coordinated, and reciprocal adaptation of mother and child behaviors has been defined as synchrony. Synchrony has often been operationalized by matched affective behaviors in gaze, vocal, and facial expression (Feldman and Eidelman 2007; Feldman et al. 2010). It supports predictability and familiarity between interacting partners, which is important for dyadic attachment and regulation (Bell 2020; Leclère et al. 2014). Self-regulation is one’s ability to manage their emotions and behaviors in order to meet the demands of the situational context (Calkins, 2011). Higher mother-child synchrony has been related to better child self-regulation (Feldman et al., 1999; Hammer et al., 2019), whereas a lack of synchrony in parent-child interactions has been associated with prolonged dyadic disorganization and children’s behavioral and temperamental difficulties (Leclère et al., 2014).

When parents and children attune to each other’s signals and respond predictably, patterns are formed that lay the foundation for homeostatic rhythms that promote child self-regulation development (Feldman 2007). This is often operationalized as contingency (Harrist and Waugh 2002) or the temporally dependent sequence or pairing of behaviors (e.g., maternal autonomy support and child persistence) that occur during parent-child interaction (Harrist and Waugh, 2002). Similar to synchrony, contingency also supports the predictability and familiarity of dyadic exchange. However, instead of emphasizing the matching of parent and child affective, behavioral, or physiological states across time, contingency analyses focus on specific lead-lag relations or behavioral sequences that may be salient for child development (e.g., child angry outburst followed by maternal soothing).

The capacity to regulate affective, behavioral, and bio-physiological states, and recover from distress emerges early in life (Tronick and Beeghly 2011). It is the parent’s primary responsibility to recognize, understand, and respond sensitively and contingently to the child’s state (co-regulation) since the child’s self-regulation ability is limited in infancy (Harrist and Waugh, 2002; Calkins, 2011). Maternal contingent responsiveness to their child has been associated with the child’s attempts to elicit social responses from the mother (Mcquaid et al., 2009), suggesting that consistent maternal responses may lay the foundation for the child’s need to develop and experience a reliable interaction with their caregivers. Furthermore, consistent parent responses to child behavior may be important for child self-regulation development (Brophy-Herb et al., 2011; Schueler and Prinz, 2013). Maternal sensitivity is associated with less frustration and anger but better regulative abilities in the child, while intrusive behavior and mental representations tinged with anger are associated with more anger and reduced regulative abilities in the child (Feldman et al., 2011a). Therefore, the parent should have the ability to regulate their own distress and to avoid a mutual transfer of distress (DiLorenzo et al., 2021). Difficulties in regulation have been associated with the child’s risk of developing psychopathology and higher levels of child externalizing and internalizing problems (Compas et al. 2017).

Borderline personality disorder (BPD) is characterized by emotion dysregulation, instability in self, and interpersonal difficulties, and it has a prevalence of 1.7% in the general population (Gunderson et al., 2018). Symptoms also impact parenthood: mothers with BPD are more likely to engage in maladaptive interactions with their child involving less reciprocal dyadic behavior (Bonfig et al. 2022; Florange and Herpertz 2019). Studies suggest less positive affect in mother and child and more child gaze aversion (White et al. 2011). Other characteristics of BPD like rejection hypersensitivity, alterations in emotion recognition, reduced self-worth and trust, and difficulties in understanding others’ mental states and emotions might also play a role in disrupted mother-child interactions (Gunderson et al. 2018; McLaren et al. 2022). Due to emotional dysregulation, coping with distress is especially difficult for individuals with BPD and may impede mothers’ abilities to adaptively and sensitively respond to their children and co-regulate their distress. Interestingly, although Kiel et al. (2011) did not find group differences between mothers with high and low BPD symptoms in their initial responses to their children’s distress, mothers with BPD showed less and delayed positive affect in response to child distress and reacted increasingly insensitively, the longer the distress lasted. In other works, mothers with BPD showed more intrusive behaviors in a reunion/repair phase after stressful situations, while their children expressed less self-regulatory behaviors (Apter et al., 2017). Therefore, not being able to offer synchronous mother-child interactions may impair their offspring’s ability to build adaptive regulative skills. Consistent evidence suggests that maternal psychopathology may increase children’s risks for mental health and regulative difficulties, for example, through an indirect effect of maternal emotion dysregulation (Gratz et al. 2014; Macfie and Swan, 2009).

In summary, the concept of synchrony and regulative behaviors in response to distress is barely understood in mothers with BPD and their children. Many studies use macro-coding to make global characterizations of the quality of mother-child interactions; however, micro-coding of verbal and non-verbal behavior utilizing a moment-to-moment timescale may offer a more dynamic perspective of how parents and children are responding to one another in real time. Taking this approach may shed more light on the importance functional, reciprocal, and regulated interaction has for the quality of the mother-child relationship (Feldman, 2007), maternal satisfaction (Atzil et al., 2011), and child self-regulation development (Beeghly and Tronick, 2011), especially in individuals who are burdened by mental disorders (Florange and Herpertz, 2019). In the present study, we analyzed behavioral synchrony and child and maternal regulative attempts during a frustration-inducing paradigm as a possible trigger of distress to detect underlying mechanisms of disrupted mother-toddler interaction in BPD. We expected (i) a reduced proportion of synchronous states and (ii) differences in maternal and child regulative behaviors (especially less consistent and effective maternal responses) during mother-toddler interactions in dyads with BPD compared to healthy control dyads; we hypothesized that (iii) both dyadic regulative behaviors and synchrony would show effects on child externalizing and internalizing behaviors. Finally, we explored associations of synchrony with (iv) BPD symptom severity to estimate the effects of the disorder on synchrony and with (v) the overall quality of mother-child interaction to further characterize how this micro-coded measure is related to a macro-coded assessment of the interactional context.

## Methods

### Participants

This sample consisted of 25 mothers with a current diagnosis of BPD (≥ 5 BPD DSM-IV criteria) and their 18- to 36-month-old toddlers. Originally, 27 mothers with BPD participated, but two mothers with BPD had to be excluded due to technical problems with their videotaped session. Additionally, 29 healthy mothers with no current or lifetime psychiatric diagnosis (healthy controls; HC) participated with their toddlers (see Table 1 and Supplementary Table 1 for group characteristics). General exclusion criteria were maternal age less than 18 or greater than 50 years, alcohol or drug (nicotine excluded) dependence over the last 24 months, severe medical illness including neurological disorders and organic brain damage, mother and toddler not living together, current pregnancy, and current breast-feeding. Some mothers with BPD had comorbid diagnoses (posttraumatic stress disorder: *n* = 6; major depression: *n* = 9; anxiety disorder: *n* = 7; obsessive-compulsive disorder: *n* = 2). Seven mothers with BPD took psychotropic medication (SSNRI: *n* = 2; SSRI: *n* = 4; methylphenidate: *n* = 1). Toddlers had no mental disorder in their reported medical history. Participants were recruited through regional in- and outpatient facilities, flyers for example in child care facilities, and online platforms.

**Table 1:** Group characteristics for mothers with borderline personality disorder (BPD) and healthy mothers (HC) and their children. *BSL* borderline symptom severity, *CIB-score* overall quality of mother-child interaction, *CBCL* child internalizing and externalizing problems. This data has been published in Bonfig et al. (2022)

	**BPD**		**HC**		***t/****χ*^2^_***df***_	***p***
	***M***	***SD***	***M***	***SD***		
**Age (years)**	30.6	7.2	31.9	4.9	− 0.79_41.48_	.436
**Age child (month)**	25.9	6.8	27.1	6.1	− 0.72_52_	.474
**Girls/boys**	14/11		15/14		0.10_1_	.753
**IPDE symptoms**	6.3	1.0	0	0	n.a.	n.a.
**BSL**	2.4	0.7	1.1	0.2	8.75_27.60_	< .001
**CIB-score**	2.8	0.8	3.5	0.4	− 3.52_32.51_	.001
**CBCL**						
**Internalizing**	10.0	5.4	5.0	3.8	3.79_42.18_	< .001
**Externalizing**	13.9	5.6	9.0	5.5	3.32_52_	< .001

### Experimental protocol

Participants were screened via telephone for exclusion criteria, participated in an onsite diagnostic interview (see Additional measures), and attended a laboratory session with their toddler at the University Hospital of Heidelberg (03/2019–07/2021). Each participant was informed about the study protocol and gave written informed consent. In an adapted 5-min toy removal paradigm (TR-paradigm; LAB-TAB, Goldsmith et al. 1995; Feldman et al. 2011b), mother and toddler were seated opposite each other at a table. The toddler received an attractive toy phone from the mother and could play with it for one minute (preTR). Following an audio signal, the mother took the toy away and placed it within the toddler’s view but outside their reach for the next 2 min (TR). Then, the mother gave the toy back for another 2 min (postTR). The mother was instructed to interact with the toddler as they would normally do during the whole paradigm.

### Coding

The videotaped interactions during the TR-paradigm were micro-coded on a computerized system (Interact, Mangold International GmbH, version 18.7.7.17). The variables *gaze*, *affect*, and *vocalization* were coded separately for mother and toddler using a timed-event sequential continuous sampling (Feldman 2014). In accordance with previous work (Feldman 2007), synchrony was defined by the proportion of time mother and toddler simultaneously looked at each other and showed positive affect during the session, as a percent (Feldman and Eidelman 2007; Feldman et al. 2011b). Synchrony was extracted for the preTR, TR, and postTR phases.

Additionally, toddler and mother regulative behaviors (i.e., child self-regulation, child seeking mother, maternal co-regulation) during the TR phase were coded (Feldman 2014; Hirschler-Guttenberg et al. 2015). Each category represented the proportion of time spent engaging in each code during the TR phase as a percent. More information on the coding procedure, variables, and composite scores can be found in Supplementary Information.

To analyze dyadic contingencies, a composite score for a *child’s negative emotionality* summarized the frequency of their protest behavior, negative affect, and negative vocalization. Dyadic contingency was operationalized as the likelihood that a criterion behavior was followed by a target behavior within a lag of 3s based on the full length of the previous event during the interaction (Beebe et al. 2016). New contingent events with (1) *child’s negative emotionality followed by maternal co-regulation* (consistency) and (2) *maternal co-regulation followed by synchrony* (effectiveness) were calculated as follows: (1) frequency of child negative emotionality followed by maternal co-regulation divided by all instances of child’s negative emotionality, and (2) maternal co-regulation followed by synchrony divided by all instances of maternal co-regulation, respectively (Apter-Levi et al. 2014; Lobo and Lunkenheimer 2020).

### Additional measures

All mothers were screened by I. S. (MD) for axis-I disorders using the German versions of the International Neuropsychiatric Interview (M.I.N.I.; Sheehan et al. 1998) and for BPD using the International Personality Disorder Examination (IPDE; Loranger et al. 1994). Additionally, borderline symptom severity (Borderline Symptom List; BSL; Bohus et al. 2007) and child internalizing and externalizing problems (Child-Behavior Checklist for Ages 1½-5; CBCL; Achenbach and Rescorla 2000) were assessed with questionnaires (see Table 1 and Supplementary Table 2). The overall quality of mother-toddler interaction was assessed by behavioral observation in a previous free-play situation, which was macro-coded for maternal, toddler, and dyadic behavior using the well-validated “Coding Interactive Behavior” manual (CIB; Feldman 1998). A total score was calculated to estimate the overall positive quality of mother-toddler interactions, with lower values indicating a less positive quality and higher values indicating a more positive quality (Feldman 1998) (for more information on the method and data of the sample, please see Bonfig et al. (2022)).

### Data analyses

Data was exported from Interact (Mangold International GmbH) and analyzed using IBM SPSS Statistics 28.0 (IBM, Armonk, NY). Independent *t*-tests for continuous variables and *χ*^2^ tests for categorical variables were used to analyze group differences in behavioral, questionnaire, and demographic data. Synchrony was analyzed by using a repeated-measure analysis of variance (rmANOVA) with group (BPD, HC) as a between-subjects factor and phase (preTR, TR, postTR) as a within-subject factor. Spearman’s correlation with Bonferroni correction for multiple testing was used to analyze associations of synchrony with the CIB total score and the BSL for dyads with BPD only. Since data on regulative behaviors and contingencies were not normally distributed, Mann-Whitney *U* tests were used to compare dyads with and without BPD for significant differences. To analyze the effects of synchrony and regulation in mother-toddler interaction on toddler’s behavior in dyads with BPD, two regression analyses were conducted each examining (1) synchrony and (2) the sum of mother and child regulative behaviors, respectively, as predictors of children’s internalizing and externalizing problems. Results were considered to be significant at *p* < .05. Partial eta squared (*η*_*p*_^2^) was used as a measure of effect sizes.

## Results

### Synchrony and regulative behaviors

**Fig. 1:**
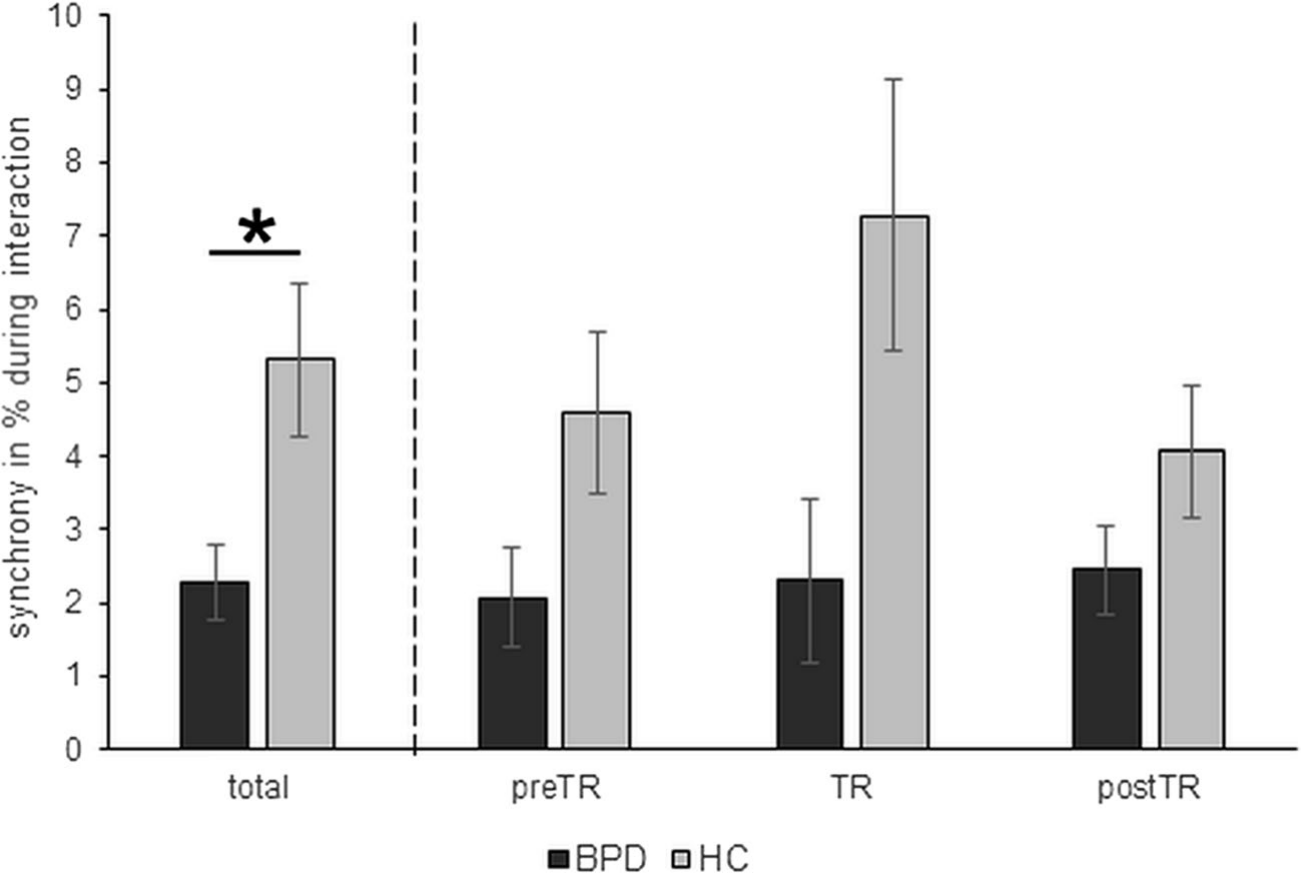
Dyadic synchrony. There was a significant group difference in the total amount of synchrony (% during whole interaction) between dyads with borderline personality disorder (BPD) and healthy controls (HC). Additionally, depicted is the amount of synchrony during the three paradigm phases: baseline (preTR), frustration induction (toy removal; TR), and after TR (postTR)

The rmANOVA showed a significant group effect (*F*_1,52_ = 5.94, *p* = .018, *η*_*p*_^2^ = .10; Fig. 1); dyads with BPD demonstrated lower levels of synchrony compared to HC. There was no significant effect for condition (*F*_2,104_ = 1.72, *p* =. 194, *η*_*p*_^2^ = .03) or a group × condition interaction (*F*_2,104_ = 1.73, *p* = .195, *η*_*p*_^2^ = .03). Correlation analyses revealed a significant association of synchrony with the CIB total score (*r* = 0.45, *p* = .025) and with borderline symptom severity (BSL; *r* = − 0.46, *p* = .020) for dyads with BPD (Bonferroni-adjusted threshold value for significance: *p* = .025).

**Fig. 2:**
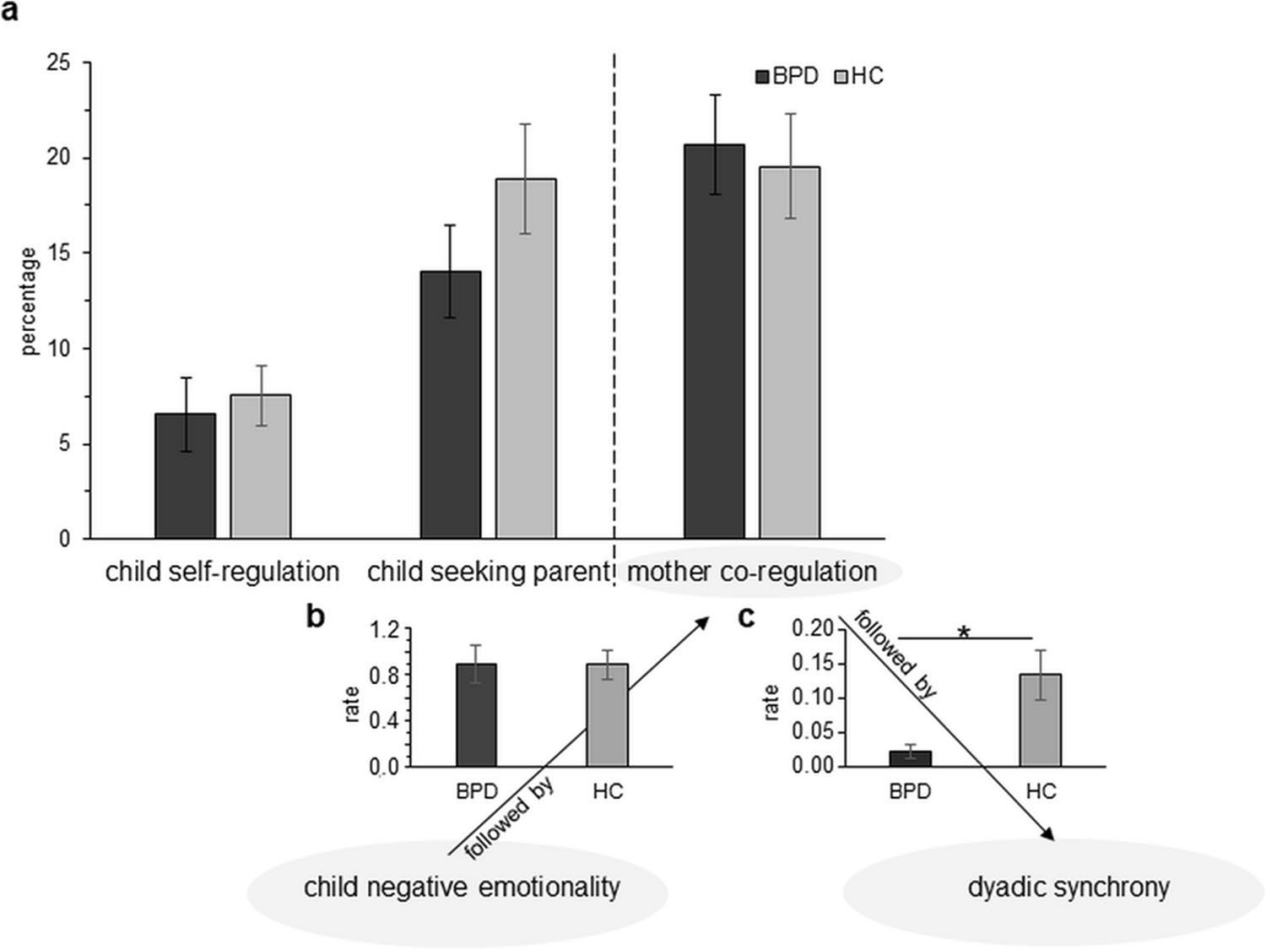
Regulative behaviors during frustration (TR) and dyadic contingencies. **a** There were no significant differences in the amount of maternal and child regulative behaviors between groups. **b** Groups did not differ in the rate with which mothers responded with co-regulation to children’s negative emotionality, but **c** the rate with which maternal co-regulation was followed by dyadic synchrony was lower in dyads with BPD compared to HC. BPD borderline personality disorder, HC healthy control. Arrows visualize contingent events

During TR, there was no significant group difference in child self-regulation (*U* = 362.50, *p* = 1.000), child seeking parent (*U* = 419.00, *p* = .327), or mother co-regulation (*U* = 346.00, *p* = .775), nor in the total sum of regulative behaviors across all conditions (*U* = 373.00, *p* = .855) between dyads with and without BPD (see Fig. 2a).

### Contingency in maternal response and co-regulation

Groups did not differ in the frequency (*U* = 271.50, *p* = .110) of children’s negative emotionality. There was no significant group difference in the number of times the mother responded with co-regulation to the child’s negative emotionality relative to all instances of the child’s negative emotionality (*U* = 271.00, *p* = .492; subsample of the group with BPD (*n* = 22; 88%) and HC (*n* = 22; 79%) because not all toddlers showed negative emotionality; see Fig. 2b). However, significantly fewer events of maternal co-regulation behaviors were followed by dyadic synchrony relative to all instances of maternal co-regulation in dyads with BPD compared to HC (*U* = 461.50, *p* = .014; see Fig. 2c).

### Prediction of child internalizing and externalizing problems

In the group with BPD, the total amount of maternal and child regulative behaviors was a statistically significant predictor of child externalizing problems with a moderate goodness-of-fit (adjusted *R*^2^ = 0.17, *F*_1,23_ = 6.03, *p* = .022, *β* = − 0.46). Lower regulation in the dyad during a frustrating situation was associated with more child externalizing problems. The amount of maternal and child regulative behaviors was only a trend-level predictor of child internalizing problems (adjusted *R*^2^ = 0.10, *F*_1,23_ = 3.54, *p* = .072). Synchrony did not predict child internalizing or externalizing problems (externalizing: adjusted *R*^2^ = − 0.04, *F*_1,23_ = 0.15, *p* = .702; internalizing: adjusted *R*^2^ < − 0.01, *F*_1,23_ = 0.98, *p* = .331).

## Discussion

This is the first study examining behavioral dyadic synchrony and regulation on a micro-level and relating it to macro-behavior in mothers with BPD and their toddlers. The study revealed three major findings: First, mothers with BPD and their toddlers showed less synchrony during interaction compared to healthy dyads. Second, there were no significant differences between dyads with and without BPD in the total amount of regulative behaviors mothers and children used during a frustration-inducing event or in the strength of the contingency between toddlers’ negative emotionality and mothers’ following co-regulative attempts. However, maternal co-regulative attempts were less often linked to subsequent synchrony between mothers with BPD and their toddlers. Third, lower regulation in the dyad during frustration predicted child externalizing problems in dyads with BPD.

We were able to confirm our first hypothesis of reduced synchrony in dyads of mothers with BPD and their toddlers. Lower synchrony levels characterize interactive disruptions on a micro-behavioral level with a reduced matching of mutual gaze and positive affect. The concept of dyadic parent-child synchrony has not been investigated in BPD so far; however, less positive maternal affect and more child gaze aversion have been observed previously (White et al. 2011). Results are in line with prior findings of disrupted social interaction in BPD and could relate to social perceptual biases and difficulties in emotion recognition and processing, social cognition, and empathy (Herpertz et al. 2018; Schneider et al. 2020). Our analyses also showed that the more severe the disorder was, the less synchronous interactions appeared. This means that more severely affected mothers may experience more difficulties in mother-child interactions and may need more support. These findings are in line with results from our previous study showing impaired overall quality of mother-toddler interaction in mothers with BPD (Bonfig et al. 2022). The interactional disturbances could have a negative impact on relationship quality, making mother-child interactions particularly stressful and unrewarding for the mother. The association of higher synchrony with a better overall quality of the mother-toddler interaction (CIB values) in the group with BPD demonstrates that our operationalization of synchrony is a relevant measure for mother-child interaction. It also underlines the importance of engaged, sensitive matching of behaviors and emotional attunement for positive mother-child relationship quality, attachment, and child regulation (Bell 2020; Leclère et al. 2014). Our results also stress that challenges to adaptive mother-child interaction lie not only within mothers with BPD or their children but within the dyad maintaining mutual coordination and flow of behaviors (Florange and Herpertz 2019; Harrist and Waugh 2002). Therapeutic work emphasizing mother-child interaction is therefore particularly important. Synchrony might not only qualify as a marker for dyadic progress, but also offer patients a helpful concept for changes on a behavioral level. Psychoeducation could help parents understand the concept of synchrony, and video-intervention therapy could help them identify their micro-level patterns. Reinforcing positive aspects and changing negative ones through cognitive and behavioral work could improve attunement to their child (Riva et al. 2016). Thus, breaking this vicious cycle of difficult relationship experiences and dysregulation in the context of interactions between mothers with BPD and their children should be the focus of intervention efforts (Leclère et al. 2014). To date, few treatment programs exist specifically for parents with BPD; they offer modified dialectical behavior therapy, home visits, and group trainings (Florange and Herpertz, 2019; May et al., 2023). Such a program has been piloted by Renneberg & Rosenbach (2016) and is currently tested in a multicenter study (Rosenbach et al. 2022). An understanding of micro-level patterns could well complement these programs and require further empirical support.

Contrary to our second hypothesis, we did not find reduced or increased maternal or child (co-)regulative attempts. This shows that dyads with BPD were fundamentally capable of expressing regulative behaviors during short periods of toddler distress. However, the stress load in our paradigm might have been too mild (2 min) to represent abnormalities more distinctly, as Kiel et al. (2011) were able to show differences between inconspicuous initial responses and increasing abnormalities as task time progressed. Interestingly, mothers with and without BPD responded equally often with co-regulatory attempts to children’s negative emotionality. However, maternal co-regulatory attempts were significantly less likely to be followed by matched dyadic synchrony in dyads with BPD. This could indicate a reduced attunement to the child and diminished effectiveness of maternal co-regulation in supporting adaptive parent-child interactions in dyads with BPD. Difficulties in successfully repairing interactions after broken cooperation or exclusion have been reported previously for patients with BPD (King-Casas et al. 2008; Reinhard et al. 2021). Reasons could be an inappropriate matching of timing and/or content of co-regulatory attempts, deviant maternal perception of and response to children’s emotional and mental states (Apter et al. 2017; Elliot et al. 2014; Gunderson et al. 2018), or an overall strained interaction and relationship so that co-regulatory attempts have little influence on dyadic quality (Elliot et al. 2014). Maternal emotion dysregulation might have also had a negative effect on the child’s emotion regulation abilities and disturbed the dyadic interaction (Gratz et al. 2014). Lastly, child behaviors like model learning and dynamic and reciprocal influences between mother and child through biological and behavioral attunement processes are likely to play an important role here (Di Lorenzo et al., 2021).

Supporting our third hypothesis, reduced dyadic regulation predicted more child externalizing problems in dyads with BPD. Maternal co-regulation, but also child self-regulation, is associated with higher quality mother-child interactions (Beeghly and Tronick 2011). Inadequate co-regulation might compromise the child’s development of building a repertoire of behavioral, cognitive, emotional, and biological strategies for regulation (Beeghly and Tronick 2011; Compas et al. 2017), and higher levels of child externalizing problems have been reported (Olson et al. 2017). Our results confirm this also for toddlers of mothers with BPD. Surprisingly, associations for internalizing problems were only trend-level significant. It could be that dyadic regulatory processes are particularly salient for externalizing problems in infancy or that mothers with BPD may have difficulty identifying internalizing problems in their toddlers.

Synchrony was unrelated to child externalizing or internalizing problems, though prior work in childhood has found evidence that higher child behavior problems may disrupt and reduce synchronous mother-child interactions (Im-Bolter et al. 2015). One explanation for this result may be that synchrony operates differently within dyads with BPD: given maternal dysregulation in BPD, opportunities for toddlers’ attunement to maternal signals may be limited and may vary in length before disruptions occur.

## Limitations

The study is limited by the small sample size and the inclusion of only mothers in this hard-to-recruit sample of patients with BPD. Results need to be confirmed in larger samples that include fathers and different age and racial/ethnic groups to generalize statements about the effects. Also, the effects of comorbidities need to be analyzed in larger samples. The paradigm was relatively short, so not all altered behaviors might have been captured. The coding system does not allow us to depict all biobehavioral complexities of dyadic processes, and in the future, temporal relations and the qualitative content need to be better captured.

## Conclusion

BPD symptomology may reduce the abilities of mother-child dyads for coordinated and attuned dyadic behavior, which we demonstrated by finding reduced synchrony in the interaction of mothers with BPD and their toddlers. Low synchrony was associated with higher BPD symptom severity and reduced overall interaction quality in BPD. Though mothers with BPD may recognize and try to respond to instances of negative emotionality in their toddlers during interaction, BPD symptomology may reduce the effectiveness of mothers’ attempts to attune to their toddler’s needs. Regulative behaviors in dyads with BPD were negatively associated with child externalizing behaviors, which could indicate adverse effects for toddlers’ development. An emphasis on synchrony and regulative behaviors may be an important therapeutic target for parenting programs in mothers with BPD and their children, and future studies are needed to investigate this direction of research.

## Supplementary information

ESM 1 (PDF 142 KB)
