# Supplementary material for: Microsocial analysis of dyadic interactions with toddlers and mothers with borderline personality disorder
Source: Arch Womens Ment Health. 2023 Jul 12;26(5):589–97. doi: 10.1007/s00737-023-01346-9 (PMC10491556; doi:10.1007/s00737-023-01346-9)
Supplement: Supplementary file 1 — ESM 1 (PDF 142 KB) [file 737_2023_1346_MOESM1_ESM.pdf]

Supplementary Information

Title: Microsocial analysis of dyadic interactions with infants and mothers with borderline personality disorder

Journal: Archives of Women's Mental Health

Authors: Isabella Schneider[a]*, Anna Fuchs[b], Sabine C. Herpertz[a], Frances M. Lobo[c]

[a] Department of General Psychiatry, Center for Psychosocial Medicine, Heidelberg University Hospital, Voßstr. 4, 69115 Heidelberg, Germany

[b] Department of Child and Adolescent Psychiatry, Center for Psychosocial Medicine, Heidelberg University Hospital Blumenstr. 8, 69115 Heidelberg, Germany

[c] Department of Psychology, The University of North Carolina at Greensboro, 294 Eberhart Building, Greensboro, NC 27402, USA

* Corresponding author:

Dr. Isabella Schneider, Department of General Psychiatry

Center for Psychosocial Medicine, Heidelberg University

Voßstr. 4, 69115 Heidelberg, Germany

Tel. +49-6221-5636601

isabella.schneider@med.uni-heidelberg.de

ORCID Identifier: 0000-0002-1179-3312

Additional group characteristic

	BPD		HC	
	M	SD	M	SD
**School education in years**	10.8	1.9	12.0	1.6
**Current occupation**				
Yes	11		16	
None	9		1	
Parental leave	5		12	
**Current relationship**				
Yes	21		26	
No	4		3	
**Currently in treatment**				
Inpatient	1		0	
Outpatient	15		0	
None	9		29	

Supplementary table 1. Additional group characteristic on school education, current occupation, current relationship and treatment situation in mothers with borderline personality disorder (BPD) and healthy mothers (HC).

Additional information on measures

**Measure**	**Reference**	**Description**	**Cronbach's alpha**
**Child Behavior Checklist** (1.5-5; CBCL)	Achenbach T, Rescorla L (2000) Child Behavior Checklist 1 ½–5 (German Version). Burlington: University of Vermont	A measure with 100 items for assessing children's internalizing and externalizing problems, which is validated for use at this developmental stage (1.5-5 years old) with good test-retest reliability and inter-rater reliability. Please see reference for more information on the reliability and validity.	.92 (BPD: 0.91; HC: 0.90)
**Borderline Symptom List** (BSL)	Bohus M, Limberger MF, Frank U, Chapman AL, Kühler T, Stieglitz R-D (2007) Psychometric properties of the borderline symptom list (BSL). Psychopathology 40:126-132. DOI: 10.1159/000098493	A self-rating questionnaire with 95 items with good psychometric properties (internal and test-retest reliability). Please see reference for more information on the reliability and validity.	0.98 (BPD: 0.98; HC: 0.84)

Supplementary table 2. Additional information on the Child Behavior Checklist and the Borderline Symptom List including Cronbach's alpha in this sample.

Coding procedure, variables and composite scores

**GAZE (coded separately for mother and child)**	
Towards the room/other objects than the toy	Mother or child looks towards the room/other objects than the toy
Towards the toy	Mother or child looks towards the toy
Towards the mother/child	Mother or child looks at their interacting partner
**AFFECT (coded separately for mother and child)**	
Negative	Mother or child show clear signs of anger, fear, or impatience
Neutral	Mother or child exhibit contentment, warm or neutral affect
Positive	Mother or child exhibit clear signs of joy, happiness, or enthusiasm
**VOCALIZATION (coded separately for mother and child)**	
Negative	Screaming, yelling, devaluating sounds
Speech	Speaking/vocalizing in a neutral/content tone
Positive	Laughing, giggling
None	No vocalization
**CHILD BEHAVIOR**	
Protest behavior	Behaviors that indicate defiance or objection, e.g. such as trying to get out of chair, hitting, kicking, moving uncomfortably in chair
Self-regulation	Self-regulation behaviors including gaze aversion from the stressor (toy), physical or verbal self-soothing, solitary substitutive play
Seeking mother	Behaviors seeking the mother including physical proximity-seeking, engaging the mother for distraction, asking for help
**MATERNAL BEHAVIOR**	
Co-regulative behavior	Behavior in which the parent is trying to help the child regulate distress including physical and verbal comfort, diverting talk and play, emotional and cognitive reflection of the situation
**COMPOSITE SCORES**	
Dyadic synchrony	Proportion of time of simultaneous gaze towards mother/child and positive affect of mother and child
Child's negative emotionality	Frequency of child events including protest behavior, negative affect, and negative vocalization
Maternal consistency	Frequency of child negative emotionality followed by maternal co-regulation divided by all instances of child's negative emotionality
Maternal effectiveness	Frequency of maternal co-regulation followed by synchrony divided by all instances of maternal co-regulation

Supplementary table 3. Coded variables

Coding procedure

Coding was conducted by two independent coders (bachelor students of psychology under the supervision of I.S.), who were trained to 90% agreement and blind to groups and all other information. For interrater reliability, 13 video-taped interactions (24%) were rated by two coders and reliability kappas for all variables averaged 0.87 over all videos (range = 0.83-0.91). The remaining video-taped interactions were only coded by one coder.

	**Kappa**
**Gaze**	
Child	0.88
Mother	0.80
**Affect**	
Child	0.85
Mother	0.86
**Vocalization**	
Child	0.84
Mother	0.82
**Protest behavior**	0.87
**Child self-regulation**	0.92
**Child seeking mother**	0.93
**Maternal co-regulation**	0.93

Supplementary table 4. Interrater reliability for the different variables
